# Reproductive health indicators of fishes from Pennsylvania watersheds: association with chemicals of emerging concern

**DOI:** 10.1007/s10661-014-3868-5

**Published:** 2014-06-17

**Authors:** V. S. Blazer, D. D. Iwanowicz, H. L. Walsh, A. J. Sperry, L. R. Iwanowicz, D. A. Alvarez, R. A. Brightbill, G. Smith, W. T. Foreman, R. Manning

**Affiliations:** 1 Fish Health Branch, Leetown Science Center, US Geological Survey, 11649 Leetown Road, Kearneysville, WV 25430 USA; 2 Columbia Environmental Research Center, US Geological Survey, 4200 New Haven Road, Columbia, MO 65201 USA; 3 Pennsylvania Water Science Center, US Geological Survey, 215 Limekiln Road, New Cumberland, PA 17070 USA; 4 Pennsylvania Fish and Boat Commission, 1601 Elmerton Avenue, Harrisburg, PA 17106 USA; 5 National Water Quality Laboratory, US Geological Survey, P.O. Box 25585, Denver, CO 80225 USA; 6 Pennsylvania Department of Environmental Protection, Rachel Carson State Office Building, 400 Market Street, Harrisburg, PA 17105 USA

**Keywords:** Smallmouth bass, White sucker, Testicular oocytes, Plasma vitellogenin, Reproductive endocrine disruption

## Abstract

Fishes were collected at 16 sites within the three major river drainages (Delaware, Susquehanna, and Ohio) of Pennsylvania. Three species were evaluated for biomarkers of estrogenic/antiandrogenic exposure, including plasma vitellogenin and testicular oocytes in male fishes. Smallmouth bass *Micropterus dolomieu*, white sucker *Catostomus commersonii*, and redhorse sucker *Moxostoma* species were collected in the summer, a period of low flow and low reproductive activity. Smallmouth bass were the only species in which testicular oocytes were observed; however, measurable concentrations of plasma vitellogenin were found in male bass and white sucker. The percentage of male bass with testicular oocytes ranged from 10 to 100 %, with the highest prevalence and severity in bass collected in the Susquehanna drainage. The percentage of males with plasma vitellogenin ranged from 0 to 100 % in both bass and sucker. Biological findings were compared with chemical analyses of discrete water samples collected at the time of fish collections. Estrone concentrations correlated with testicular oocytes prevalence and severity and with the percentage of male bass with vitellogenin. No correlations were noted with the percentage of male sucker with vitellogenin and water chemical concentrations. The prevalence and severity of testicular oocytes in bass also correlated with the percent of agricultural land use in the watershed above a site. Two sites within the Susquehanna drainage and one in the Delaware were immediately downstream of wastewater treatment plants to compare results with upstream fish. The percentage of male bass with testicular oocytes was not consistently higher downstream; however, severity did tend to increase downstream.

**Electronic supplementary material:**

The online version of this article (doi:10.1007/s10661-014-3868-5) contains supplementary material, which is available to authorized users.

## Introduction

Indications of endocrine disruption are widespread in aquatic ecosystems worldwide (Jobling et al. 1998; Colburn and Thayer 2000; McMaster 2001; Mills and Chichester 2005; Hinck et al. 2009; Pal et al. 2010). The finding of a high prevalence of intersex in smallmouth bass *Micropterus dolomieu* in the Potomac drainage raised concern about the potential effects of exposure to endocrine-disrupting or modulating chemicals (EDC) in the Chesapeake Bay watershed (Blazer et al. 2007). Contaminants of emerging concern (CEC) include chemicals that are not currently regulated or commonly monitored in the aquatic environment such as pharmaceutical compounds (human and veterinary), natural and synthetic hormones, flame retardants, alkyl phenols, and other organic wastewater compounds (Daughton and Ternes 1999; Hotchkiss et al. 2008; Diamant-Kandarakis et al. 2009). Also, of emerging concern are newly recognized effects, such as endocrine disruption and immunosuppression, of pesticides (legacy and current use), persistent organic compounds including polychlorinated biphenyls (PCBs), and metals including arsenic and mercury. Many of these chemicals can have significant biological effects on aquatic organisms, through endocrine-modulated pathways (Klaper et al. 2006; Davey et al. 2007; Eldridge et al. 2008; Kugathas and Sumpter 2011; Martyniuk et al. 2011; Orton et al. 2011) and modulation of the immune system (Hermann and Kim 2005; Iwanowicz et al. 2005; Datta et al. 2009).

Intersex in the form of testicular oocytes (TO) was first noted in smallmouth bass (SMB) during the microscopic examination of tissues in response to fish mortalities observed in 2002–2003 in the South Branch Potomac River. Since then, mortalities have also occurred in the Shenandoah and the Monocacy Rivers, both tributaries of the Potomac River, and moderate to high prevalence of TO has been documented at these sites as well (Blazer et al. 2007; Iwanowicz et al. 2009; Blazer et al. 2010). Mortalities in the Potomac drainage have involved mature adult fishes and occurred in the spring. The location, extent, and severity of these mortalities have varied annually, and no single pathogen or water quality parameter has been identified as the cause. Rather, the numerous bacterial pathogens isolated and parasites observed suggest general immunosuppression, most likely resulting from exposure to numerous environmental stressors (Blazer et al. 2010). The co-occurrence of skin lesions and excessive mortality and signs of exposure to EDC (intersex and vitellogenin in male fish) suggest that chemicals associated with feminization of male fishes might also be associated with reduced disease resistance. Mortalities of SMB have occurred in the Susquehanna River since 2005, although they differ from those observed in the Potomac in that young-of-year, rather than adults that are involved (Chaplin et al. 2009). However, as in adults from the Potomac, there are numerous bacterial and viral pathogens (Smith et al. 2014) and parasites observed in affected individuals (Walsh et al. 2012), raising the possibility that immunosuppressive water quality conditions, including CEC, may be involved in these mortalities as well.

In 2006, the US Geological Survey’s Pennsylvania Water Science Center and Pennsylvania Department of Environmental Protection surveyed surface and groundwater sites of South Central Pennsylvania for a suite of CEC (Loper et al. 2007). The findings of that study led to the development of a second, more comprehensive assessment of the occurrence and concentrations of CEC spatially and temporally, as well as identification of potential sources at sites within the three major river drainages (Susquehanna, Delaware, and Ohio) of Pennsylvania (Reif et al. 2012). Fish were sampled at a subset of these sites to assess effects since it is recognized that biological effects-based monitoring can indicate cumulative effects of chemical exposures throughout the life of the fish, which might not be captured by discrete water samples at the time of sampling (Dubé and Munkittrick 2001; Eckman et al. 2013). Significant mixture effects have been demonstrated that are often unpredictable (Sárria et al. 2011), but environmental estrogens can be additive at environmentally relevant concentrations (Silva et al. 2002; Brian et al. 2005). Low-dose exposures at sensitive life stages can have long-term effects including immunosuppression and reproductive impairment later in life (Milston et al. 2003; Liney et al. 2005; Leet et al. 2011; Vandenberg et al. 2012).

Hence, the objectives of this study were threefold: (1) to document biological effects, specifically, prevalence and severity of TO and other indicators of reproductive endocrine disruption at sites in the three major river basins in Pennsylvania; (2) to compare these findings to chemical concentrations and potential sources and land use; and (3) to compare species responses.

## Materials and methods

### Sites

**Fig. 1:**
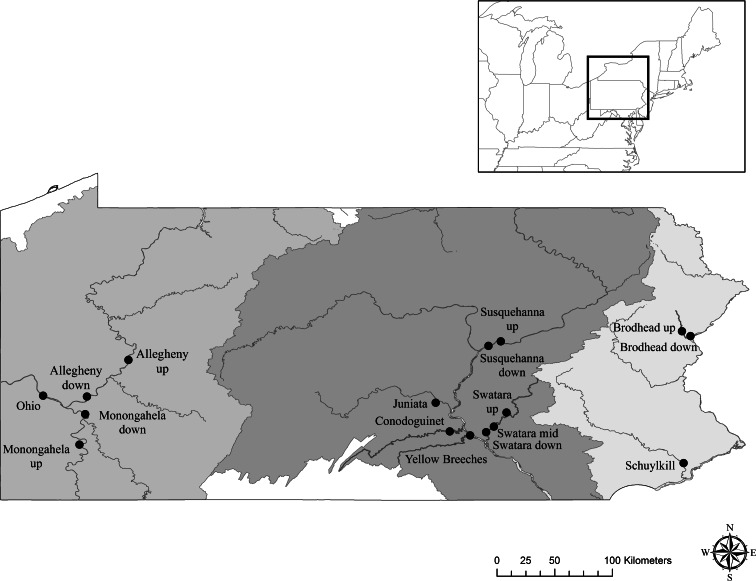
Fish collection sites in Pennsylvania drainages from 2007 to 2010

Fish were collected from a total of 16 sites within the three river drainages in Pennsylvania from 2007 to 2010 (Fig. 1). These included three sites in the Delaware River drainage, eight sites in the Susquehanna River drainage, and five sites in the Ohio River drainage. Selected sites were chosen to specifically address potential effects of specific wastewater treatment plants (WWTPs) by collecting fish upstream and immediately downstream of these plants. These included three sites on Swatara Creek, a tributary of the Susquehanna. The upper site was close to Harper Tavern, and although there are some smaller WWTPs upstream (Table 1), no major municipal facilities occur upstream. The second site was close to Hershey. Above this site is a tributary, Quittapahilla Creek, into which the city of Lebanon, the borough of Annville and Palmyra Area WWTPs, all discharge. The third site was near Hummelstown, and both the Hershey and Hummelstown WWTP discharges are upstream. Two sites sampled on the mainstem Susquehanna River were upstream and downstream of the Danville WWTP which includes effluent from a major hospital. Within the Delaware drainage, there were two sites on Brodhead Creek, upstream and downstream of a WWTP. While sampling occurred upstream and downstream of a specific WWTP, none of the sites were in headwaters, and hence in all cases, other effluent sources were present upstream. Sampling in the Ohio drainage did not focus on any particular WWTP; however, there were upstream and downstream sites on the Allegheny and the Monongahela Rivers.

**Table 1:** Locations and sampling dates of fish collections in three river drainages within Pennsylvania

Site description	Location		Sampling date
	Latitude	Longitude	
Delaware drainage			
Schuylkill River at Fall Bridge	40.008400	−75.197500	July 11, 2007
Brodhead Creek near East Stroudsburg (upstream)	41.037000	−75.210000	July 19, 2007
Brodhead Creek at Minisink Hills (downstream)	40.998500	−75.143300	July 18, 2007
Susquehanna drainage			
Conodoguinet Creek near Hogestown	40.255200	−77.018900	July 12, 2007
Susquehanna River at Danville (upstream)	40.957700	−76.621200	July 17, 2007
Susquehanna River (downstream)	40.922300	−76.717900	July 16, 2007
Swatara Creek at Harper Tavern (upstream)	40.402500	−76.577400	August 1, 2007
Swatara Creek near Hershey (mid)	40.293321	−76.674794	August 2, 2007
Swatara Creek near Hummelstown (downstream)	40.249900	−76.736200	August 16, 2007
Juniata River at Newport	40.479002	−77.128519	August 14, 2007 April 21, 2010
Yellow Breeches Creek at New Cumberland	40.224186	−76.860560	August 15, 2007
Ohio drainage			
Ohio River at Sewickley	40.533370	−80.187562	July 23, 2008
Monongahela River at North Charleroi (upstream)	40.389900	−79.859200	July 21, 2008
Monongahela River at Braddock (downstream)	40.152010	−79.904123	July 22, 2008
Allegheny River at Kittaning (upstream)	40.812600	−79.522600	August 4, 2008 April 28, 2010
Allegheny River at Oakmont (downstream)	40.527100	−79.846400	August 5, 2008

Drainage size, flow at time of fish collection, and land cover characteristics within each watershed were assessed. The number of sewage and WWTP discharges within each watershed was also documented (Table 2).

**Table 2:** Drainage size and land cover characteristics of the watersheds with fish collection sites

Sites	Drainage area (km^2^)	Discharge (m^3^/s)	Land Cover Entire watershed (%) Forest agriculture urban			Wastewater treatment plant discharges
Delaware drainage						
Schuylkill	4,903	20.70	40	38	19	435
Brodhead up	313	1.30	78	–	11	7
Brodhead down	671	2.89	70	4	19	17
Susquehanna drainage						
Conodoguinet	1,217	4.11	35	51	13	60
Susquehanna up	29,060	85.20	60	28	6	298
Susquehanna down	29,267	66.80	59	28	7	308
Swatara up	873	1.90	51	37	11	31
Swatara mid	1,251	3.82	43	41	14	51
Swatara down	1,422	4.56	40	41	17	57
Juniata	8,687	26.20	70	22	8	174
Yellow Breeches	534	3.62	52	30	17	22
Ohio drainage						
Ohio	50,505	909.00	69	17	9	1,718
Monongahela up	13,546	108.00	77	13	8	124
Monongahela down	19,003	688.00	73	15	10	433
Allegheny up	23,240	137.00	69	18	5	715
Allegheny down	29,984	163.00	68	18	7	1,122

### Fish collections

At each site, we attempted to collect at least 20 fish (10 of each sex) of two species. The rationale was to compare two species at each site that represented different food and habitat preferences. Species included SMB, a member of the Centrarchidae family, representing carnivorous, pelagic species. SMB have been shown to be a sensitive indicator species for EDC studies, expressing signs of estrogenic exposure such as TO and plasma vitellogenin (Vtg) in males (Baldigo et al. 2006; Blazer et al. 2007, 2012; Hinck et al. 2009; Iwanowicz et al. 2009). Suckers are benthic feeding, bottom-dwelling species in the Catostomidae family. White sucker (WS) *Catostomus commersonii*, a long-lived gonochoristic species (Lalancette 1975), has been used extensively in monitoring and assessment studies (Bowron et al. 2009; McMaster 2001; Miller et al. 2009) and been shown to respond to EDC exposure (Dorval et al. 2005; Woodling et al. 2006; Vajda et al. 2008). WS were not available at the sites sampled in the Ohio drainage. Redhorse sucker (RHS) *Moxostoma* species, including the golden redhorse *Moxostoma erythrurum* and the black redhorse *Moxostoma duquesnei*, are common in the Ohio drainage. These species have not been used previously as an indicator species for monitoring effects of EDC and information on microscopic gonadal development, and reagents for Vtg analyses are lacking. However, reports in the popular press of “genderless fish,” including catfishes and suckers, raised public concern. Hence, we collected gonads from these species for histological evaluation.

Within the Delaware and Susquehanna River drainages, SMB and WS were collected in the summer of 2007 (July 11–August 16). In the Ohio River drainage, SMB and RHS were collected in the summer of 2008 (July 23–August 5). All these species are spring spawners, and hence in summer, post-spawn is the least reproductively active period. However, this sampling period was chosen to represent low flow conditions. Pre-spawn SMB were compared at one site in the Susquehanna drainage (Juniata River) and one site in the Ohio drainage (upstream Allegheny) in spring 2010 (April 21–28). Exposure to agriculturally associated chemicals is likely to be highest during spring high flow periods after field application and when runoff is most likely.

Fish were collected by either barge or boat electrofishing and held in large, aerated buckets of river water until processed (less than 1 h). Fish were euthanized with tricaine methanesulfonate (Finquel, Argent Laboratories, Redmond, Washington) and bled from the caudal vein using heparinized 3-cc syringes with 23 gauge needles. Blood was placed in Vacutainer tubes (Fisher Scientific, Pittsburgh, PA) containing sodium heparin and held on wet ice until centrifuged (same day) for 10 min at 1,000 × *g* and 4 °C for plasma separation. Plasma was removed, aliquoted into cryovials, and stored at −80 °C until assayed for Vtg.

Each fish was weighed (to the nearest gram), measured (to the nearest millimeter), and observed for gross lesions and abnormalities, liver and gonad were removed and weighed to the nearest 0.1 g. Condition factor (Ktl) was calculated by the formula: ((body weight − gonad weight in grams) / length^3^ in millimeter) × 10^5^. A complete necropsy-based fish health assessment as described by Goede and Barton (1990) was completed. External abnormalities on both the body surface (red raised, eroded or mucoid lesions, black spot, leeches, cloudy eyes) and gills (small white spots, grubs, pale coloration, eroded, frayed) were recorded. Scales from the lateral body surface, above the left pectoral fin and lateral line, were removed for age determination.

### Reproductive endpoints

Pieces of gonad were fixed in Z-Fix™ (Anatech Ltd., Battle Creek, Michigan) for histological analysis. Gonadosomatic index (GSI) was calculated as follows: (gonad weight / body weight) × 100. Plasma Vtg concentrations in SMB and WS were measured using a direct enzyme-linked immunosorbent assay (ELISA) with species-specific monoclonal antibodies and were carried out at the Leetown Science Center or the University of Florida, Center for Human and Environmental Toxicology, as described by Denslow et al. (1999) and Blazer et al. (2012).

Five to seven cross sections were taken along the gonads of both male and female fish and processed for histological evaluation; provided enough tissue was present. The tissue pieces were embedded in paraffin, sectioned with a microtome at 6 μm, and stained with hematoxylin and eosin (Luna 1992). Sections were examined microscopically to confirm sex, determine the stage of gonad development, and document intersex and other microscopic abnormalities (Blazer 2002; Dietrich and Krieger 2009). Intersex in bass is observed as the presence of immature oocytes within the testes. The severity index for bass intersex (Blazer et al. 2007) is based on the number and distribution of oocytes within the testes. Focal distribution (score 1) is a single oocyte within the field of view of testicular tissue (scores determined at a magnification of × 200). Diffuse distribution (score 2) is more than one oocyte in the field of view but with no physical association with neighboring oocytes. Cluster distribution (score 3) is more than one but less than five physically associated oocytes in a field of view. Zonal distribution (score 4) is five or more physically associated oocytes or numerous clusters of oocytes within a field of view. The scores for each of five to seven cross sections were averaged, yielding the mean score for each fish which was then averaged to generate site severity values.

### Chemical analyses

Discrete water and bed sediment samples were collected at the time of fish sampling. Methodology and occurrence data are presented in Reif et al. (2012). In Spring 2010, polar organic chemical integrative samplers (POCIS) and semipermeable membrane devices (SPMDs) were deployed at the two fish collection sites to determine the presence of dissolved-phase chemicals that might occur episodically or in concentrations too low to be detected by conventional methods (Alvarez 2010). The SPMDs are designed to monitor lipid soluble or nonpolar chemicals (Petty et al. 2000), while POCIS monitor more polar or hydrophilic chemicals (Alvarez 2010). Samplers were deployed at the Juniata River site for 42 days (April 21, 2010 to June 1, 2010) and at the Allegheny River site for 41 days (April 28, 2010 to June 7, 2010). Samplers were extracted using established procedures previously described (Alvarez et al. 2009). Extracts of the POCIS were screened for total estrogenicity using the bioluminescent yeast-based estrogen screen (Sanseverino et al. 2009 as modified in Alvarez et al. 2009), synthetic and biogenic hormones by adaptation of a water method (Foreman et al. 2012), and agricultural pesticides (Alvarez et al. 2009). Extracts from the SPMDs were screened for total PCBs, polycyclic aromatic hydrocarbons (PAHs), polybrominated diphenyl ethers (PBDEs), and organochlorine pesticides (Alvarez et al. 2009). Chemicals measured in the passive samplers and their method detection limits (MDL) and reporting levels (RL) are presented in Supplementary Tables 1 and 2.

### Statistical analyses

If data were normally distributed, means were compared using Tukey’s multiple comparison test. Data not normally distributed were compared using nonparametric ANOVA followed by Dunn’s multiple comparison test to compare site medians. For upstream/downstream comparisons, a two-tailed Mann–Whitney test was performed. A Spearman’s rank correlation (rho) was used to test for the relation between TO or plasma Vtg and land cover characteristics or chemical concentrations (GraphPad InStat 3, GraphPad Software, Inc., La Jolla, CA). A significance (*p*) level of 0.05 was used for all statistical analyses.

## Results

### Smallmouth bass—morphometric and fish condition

Adult SMB were collected from six sites in the Susquehanna and one in the Delaware drainage in the summer of 2007 and five sites in the Ohio drainage in the summer of 2008. There was no significant difference between males and females for any of the morphometric endpoints or fish condition; hence, the sexes were pooled for site comparisons. Mean lengths and weights were similar among sites within the Susquehanna drainage except those collected at the Juniata site which were significantly (*p* < 0.0001) longer, heavier, and older than all other sites (Table 3). There was no significant difference when comparing only the three Swatara sites or the two mainstem Susquehanna sites upstream and downstream of WWTPs for age, length, or weight. Mean Ktl of SMB collected at the three sites on Swatara Creek was different from one another (*p* = 0.0199), with bass from upstream having a lower mean Ktl than those from the middle, while those from the downstream site were not different than either of the other two sites. The two Susquehanna sites were not different in a pairwise comparison (Table 3).

**Table 3:** Morphometric characteristics of smallmouth bass *Micropterus dolomieu* collected from river drainages of Pennsylvania in 2007–2010

Site	Season year	Sample size	Age^a^ (years)	Total length^a^ (mm)	Weight^a^ (g)	Condition factor^a^ (Ktl)^b^
Delaware drainage						
Schuylkill	Summer 2007	20	ND	248 ± 48	208 ± 128	1.24 ± 0.07
Susquehanna drainage						
Susquehanna up	Summer 2007	20	3.7 ± 1.0	283 ± 51	354 ± 231	1.41 ± 0.14
Susquehanna down	Summer 2007	21	3.4 ± 1.0	297 ± 66	440 ± 340	1.46 ± 0.11
Swatara up	Summer 2007	8	2.9 ± 0.6	262 ± 56	282 ± 236	1.35 ± 0.10
Swatara mid	Summer 2007	14	3.3 ± 0.5	266 ± 38	303 ± 210	1.47 ± 0.10
Swatara down	Summer 2007	12	4.1 ± 0.9	282 ± 30	321 ± 109	1.38 ± 0.08
Juniata	Summer 2007	20	5.6 ± 1.3	376 ± 61	884 ± 388	1.58 ± 0.20
	Spring 2010	20	ND	391 ± 64	969 ± 474	1.39 ± 0.29
Ohio drainage						
Ohio	Summer 2008	20	2.3 ± 0.6	217 ± 41	131 ± 70	1.19 ± 0.08
Monongahela up	Summer 2008	20	ND	283 ± 58	273 ± 187	1.07 ± 0.08
Monongahela down	Summer 2008	20	ND	249 ± 53	188 ± 116	1.09 ± 0.12
Allegheny up	Summer 2008	20	3.3 ± 0.6	278 ± 43	261 ± 115	1.17 ± 0.15
	Spring 2010	20	ND	317 ± 39	377 ± 126	1.12 ± 0.09
Allegheny down	Summer 2008	20	2.5 ± 1.1	239 ± 43	183 ± 84	1.21 ± 0.08

*ND* no data

^a^Data presented as mean ± standard deviation

^b^Ktl calculated by ((body weight − gonad weight) / length^3^) × 10^5^

Within the Ohio drainage, SMB from the Ohio site were the smallest fish, significantly different (*p* < 0.05) in length and weight than those from all sites except the downstream Monongahela site. In pairwise comparisons, there was no difference in SMB collected at upstream and downstream sites on the Monongahela River. However, bass from the upstream Allegheny site were longer (*p* = 0.0274), heavier (*p* = 0.0417), and older (*p* = 0.0180) than those from the downstream site (Table 3).

The SMB collected at both sites in spring 2010 were larger, although not significantly different than those collected at the same site during summer 2007. Those collected in Allegheny were shorter than those collected in Juniata (*p* = 0.0006). Weights of SMB collected at Allegheny were less than those collected at the Juniata site (*p* < 0.0001) as were condition factors (Table 3).

### Smallmouth bass—reproductive endpoints

**Fig. 2:**
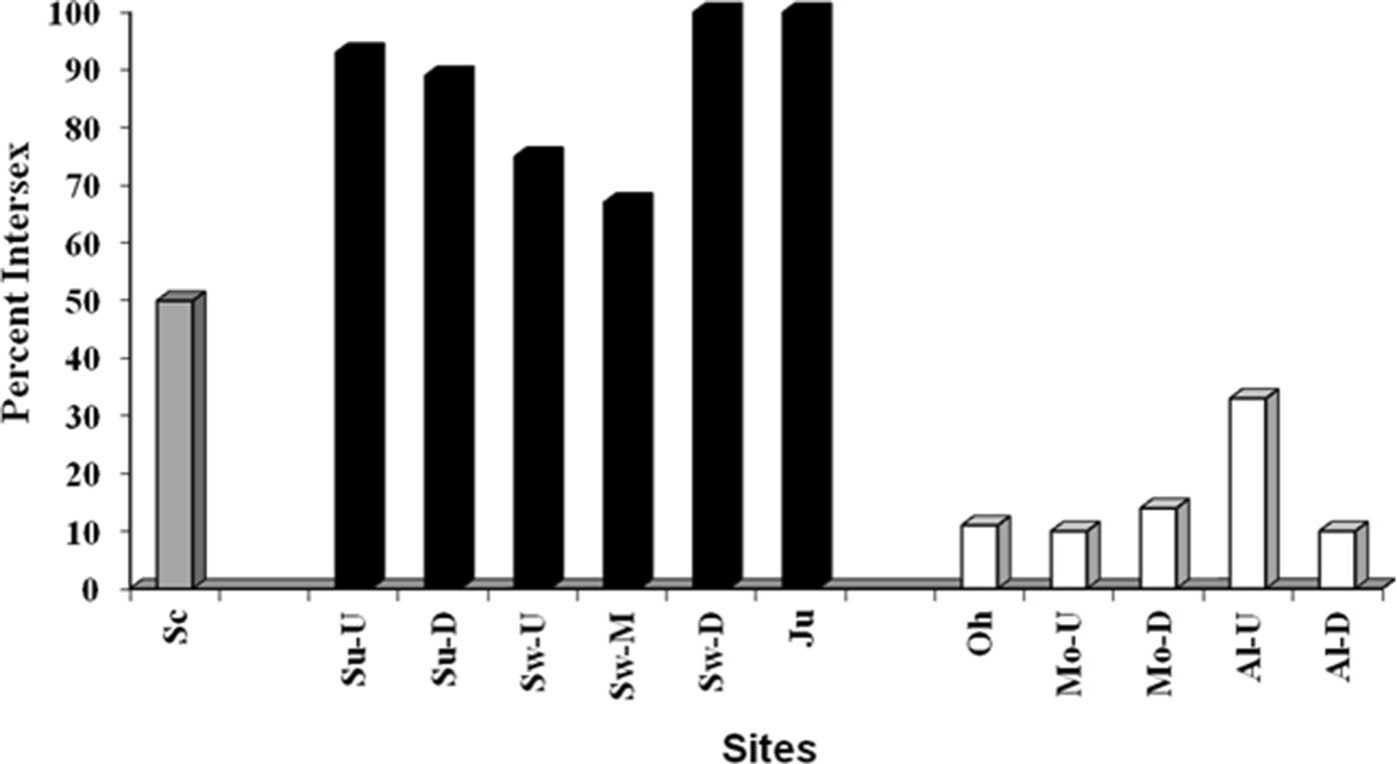
Percentage of male smallmouth bass with testicular oocytes collected in the three major river drainages in Pennsylvania in 2007–2008. One site, Schuykill River (*Sc*) in the Delaware drainage (*gray bar*); six sites, upstream (*Su-U*) and downstream (*Su-D*) on the Susquehanna River, upstream (*Sw-U*), middle (*Sw-M*), and downstream (*SW-D*) Swatara Creek and Juniatia River (*Ju*) in the Susquehanna drainage (*black bars*); Ohio River (*Oh*), upstream (*Mo-U*) and downstream (*Mo-D*) Monongahela River, upstream (*AL-U*) and downstream (*Al-D*) Allegheny in the Ohio drainage (*white bars*)

**Fig. 3:**
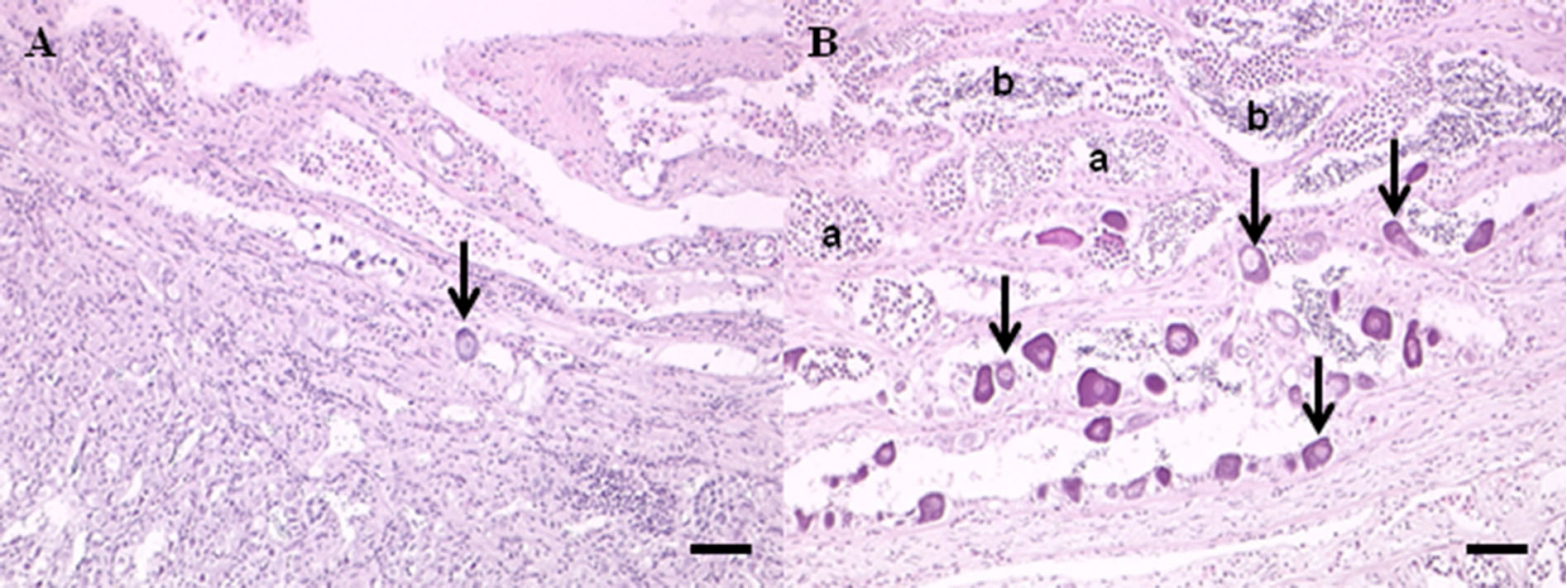
Microscopic appearance of testicular tissue in smallmouth bass and sucker species collected in the Susquehanna and Ohio drainages in 2007–2008. **a** Testicular tissue of smallmouth bass collected in the Ohio River drainage illustrating low severity of testicular oocytes. One oocyte (*arrow*) is apparent in the section. **b** Section of testes from a smallmouth bass collected in the Susquehanna River drainage. Numerous oocytes including clusters (*arrows*) are present. Multiple sperm cell stages including spermatocytes and spermatids (*a*) and spermatozoa (*b*) are present. *Scale bars* = 50 μm. Hematoxylin and eosin stain

Attempts were made to collect 10 individuals of each sex; however, that was not possible at some sites. Six or more males were collected at each site except for the upstream Swatara site at which only four were collected (Table 4). Gonad weights were low (<0.5 g) for male SMB collected in 2007–2008. This is not unexpected as fish were collected in the summer, post-spawn. Therefore, statistical analyses of GSI were not conducted. TO were observed in SMB from all drainages. In general, during the summers of 2007 and 2008, TO prevalence was lowest in the Ohio drainage, intermediate in the Delaware, and highest in the Susquehanna (Fig. 2). Intersex severity was also different between the drainages (Table 4). In the Ohio drainage, few to no oocytes were observed in individual sections of testes (Fig. 3a), while testes sections from SMB collected in the Susquehanna contained many more oocytes (Fig. 3b). When comparing all sites, there were significant differences (*p* < 0.0001) among sites. The Susquehanna upstream and downstream, Juniata, and downstream Swatara sites had a higher severity than all the Ohio sites which were all similar to one another. Testes from male bass collected at the Schuylkill, Swatara upstream, and Swatara mid-sites had mean severity ratings that were intermediate and not different from either the Ohio sites or the other Susquehanna sites (Table 4). Intersex prevalence was high in SMB collected from both sites (Juniata 67 % and Allegheny 75 %) in spring 2010. However, severity in SMB from Allegheny was significantly lower (*p* < 0.0001) than those collected in Juniata (Table 4).

**Table 4:** Reproductive endpoints of male smallmouth bass collected from three river drainages in Pennsylvania in 2007–2010

Site	Sample size	Intersex severity^a^	Mean vitellogenin^b^ (μg/mL)
Delaware drainage			
Schuylkill	10	0.6 ± 0.9 ab	166.7 ± 11.9 (9)
Susquehanna drainage			
Susquehanna up	14	1.4 ± 0.3 a	4.3 ± 1.2 (3)
Susquehanna down	8	1.8 ± 0.4 a	113.6 ± 61.8 (5)
Swatara up	4	0.6 ± 0.4 ab	0
Swatara mid	6	0.9 ± 0.7 ab	8.0 ± 3.8 (3)
Swatara down	6	2.0 ± 1.2 a	0
Juniata Summer	7	2.6 ± 0.5 a	155.4 ± 27.9 (7)
Spring 2010	9	2.0 ± 0.5 A	431.7 ± 823.5 (6)
Ohio drainage			
Ohio	9	0.1 ± 0.2 b	66.0 ± 0.0 (1)
Monongahela up	10	0.1 ± 0.2 b	37.0 ± 0.0 (1)
Monongahela down	7	0.1 ± 0.2 b	13.0 ± 0.0 (1)
Allegheny up	6	0.2 ± 0.3 b	31.0 ± 0.0 (1)
Spring 2010	12	0.3 ± 0.5 B	338.9 ± 373.5 (9)
Allegheny down	10	0.1 ± 0.3 b	36.5 ± 26.2 (2)

^a^Mean ± standard deviation. Values followed by the same lowercase letters collected during summers of 2007–2008 or uppercase letters collected in Spring 2010 were not significantly different

^b^Mean ± standard deviation of only those individuals with measurable vitellogenin. Number in parentheses indicates sample size of positive males

**Fig. 4:**
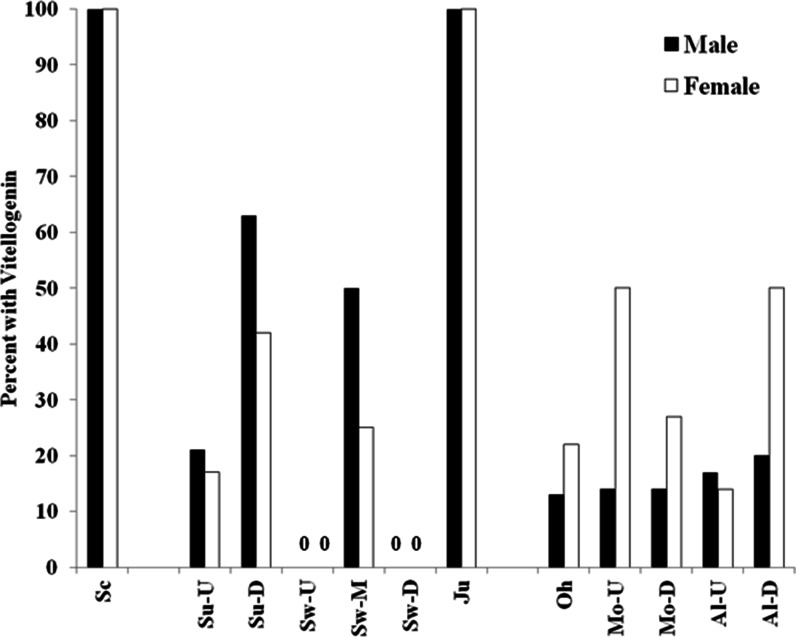
Percentage of male (*black bars*) and female (*white bars*) smallmouth bass with measurable vitellogenin collected in the three major river drainages (Delaware, Susquehanna, and Ohio) in 2007–2008. One site, Schuykill River (*Sc*) in the Delaware drainage; six sites, upstream (*Su-U*) and downstream (*Su-D*) on the Susquehanna River, upstream (*Sw-U*), middle (*Sw-M*), and downstream (*SW-D*) Swatara Creek and Juniata River (*Ju*) in the Susquehanna drainage; Ohio River (*Oh*), upstream (*Mo-U*) and downstream (*Mo-D*) Monongahela River, upstream (*AL-U*) and downstream (*Al-D*) Allegheny in the Ohio drainage

All male SMB collected in the Schuylkill River had measurable Vtg. The percent of males within the Susquehanna drainage with detectable Vtg ranged from 0 % at the Swatara upstream and downstream sites (50 % at mid-Swatara) to 100 % in the Juniata River (summer and spring). On the Susquehanna mainstem, 63 % of the SMB had measurable Vtg at the downstream site, while only 21 % had measurable amounts at the upstream site (Fig. 4). Mean concentrations, using only samples with measurable amounts, were highest in SMB collected from Schuylkill, Susquehanna downstream, and Juniata. Summer Vtg concentrations in most male SMB from the Ohio drainage were nondetectable, and so statistical analyses were not conducted. The percentage of males with Vtg was higher in the spring, and mean concentrations were about 3 and 10 times higher in the spring than in summer at the Juniata and Allegheny sites, respectively (Table 4).

A comparison of SMB collected at upstream and downstream sites on the mainstem Susquehanna showed no significant difference in TO prevalence (*p* = 1.000) or severity (*p* = 0.3705), percentage of males with Vtg (*p* = 0.0815), or mean Vtg of those individuals with measurable concentrations (*p* = 0.1429). Sites on Swatara Creek had significantly different (*p* = 0.048) TO severities with an increasing gradient from upstream to downstream. However, only the middle site had males with measurable Vtg concentrations.

Sample size for female bass was below six at the Swatara upstream, Allegheny downstream, and Allegheny upstream sites in the spring. Female SMB collected in the summers of 2007–2008 also had relatively small gonads. Mean GSI ranged from 0.23 to 0.69 with no consistent differences among drainages. There were no differences (*p* = 0.3899) in GSI among female SMB collected in the Ohio or Susquehanna drainages. Although not statistically different, it is interesting that female GSI were higher at the upstream than at the downstream sites in all cases (Table 5). The percentage of female SMB with measurable Vtg ranged from 0 to 100 %. In general, within the Susquehanna drainage, more male than female SMB had measurable concentrations of Vtg in the summer, and concentrations (Fig. 4) were similar between the sexes. In the Ohio drainage, more females than males had measurable Vtg, but only in samples from the Monongahela were the concentrations higher in the females than males (Tables 4 and 5).

**Table 5:** Reproductive endpoints of female smallmouth bass collected from three river drainages in Pennsylvania in 2007–2010

Site	Sample size	Gonadosomatic index^a^	Mean vitellogenin^b^ (μg/mL)
Delaware drainage			
Schuylkill	10	0.50 ± 0.13 b	161.5 ± 18.2 (10)
Susquehanna drainage			
Susquehanna up	6	0.33 ± 0.12 ab	9.0 ± 0.0 (1)
Susquehanna down	12	0.23 ± 0.09 a	125.8 ± 18.8 (5)
Swatara up	4	0.69 ± 0.51 ab	0
Swatara mid	8	0.36 ± 0.11 ab	11.0 ± 11.3 (2)
Swatara down	6	0.43 ± 0.08 ab	0
Juniata	13	0.50 ± 0.14 b	152.7 ± 28.5 (13)
Spring 2010	10	11.10 ± 12.90	14,092.1 ± 10,770.0 (10)
Ohio drainage			
Ohio River	9	0.68 ± 1.05 ab	17.5 ± 6.4 (2)
Monongahela up	9	0.57 ± 0.20 b	183.0 ± 163.1 (3)
Monongahela down	13	0.48 ± 0.19 b	109.3 ± 118.4 (3)
Allegheny up	13	0.45 ± 0.21 ab	27.0 ± 19.8 (2)
Spring 2010	5	6.30 ± 4.10	8,831.0 ± 2,048.0 (5)
Allegheny down	4	0.43 ± 0.06 ab	31.0 ± 28.6 (3)

^a^Mean ± standard deviation. Values followed by the same lowercase letters collected during summers of 2007–2008 were not significantly different

^b^Mean ± standard deviation of only those individuals with measurable vitellogenin. Number in parentheses indicates sample size of positive females

Plasma Vtg concentrations were higher in females than males at the two sites fish were collected in spring, a time when females would normally have high circulating Vtg levels. There was no difference in Vtg concentration of male (*p* = 0.3215) or female (*p* = 0.1651) SMB collected in spring 2010 at the Allegheny and Juniata sites (Tables 4 and 5).

### White sucker—morphometric and fish condition

WS were collected at three sites in the Delaware drainage and five sites in the Susquehanna drainage. There were size differences between males and females, and so they were analyzed separately. At the sites for which age data was available, the females tended to be older. Females also tended to be larger at most sites with the exception of the Schuylkill River site at which the males were larger. Within the Delaware drainage, female WS collected at the Schuylkill River site and the downstream Brodhead Creek site were similar in length and weight, while those collected at the upstream Brodhead Creek site were significantly shorter and lighter (*p* < 0.001). Condition factors of females were not significantly different among the three sites. A similar trend was seen in male WS with those collected at the upstream Brodhead Creek site being shorter and lighter than the other two sites (*p* < 0.0001), and condition factors were not different (Table 6). Suckers collected upstream of the WWTP on Brodhead Creek were significantly shorter and weighed less (*p* < 0.0001) than those collected downstream.

**Table 6:** Morphometric characteristics of white sucker collected from the Delaware and Susquehanna River drainages of Pennsylvania in 2007–2008

Site	Sex	Age (years)	Sample size	Total length^a^ (mm)	Weight (g)	Condition factor (Ktl)^b^
Delaware drainage						
Schuylkill	Male	ND	7	380 ± 35	778 ± 193	1.19 ± 0.03
	Female	ND	13	369 ± 31	589 ± 159	1.15 ± 0.07
Brodhead up	Male	2.6 ± 0.8	13	245 ± 68	166 ± 117	1.07 ± 0.16
	Female	2.8 ± 0.7	9	250 ± 95	224 ± 314	1.07 ± 0.11
Brodhead down	Male	2.9 ± 0.6	9	401 ± 95	642 ± 276	1.17 ± 0.05
	Female	3.7 ± 1.0	11	445 ± 85	1,060 ± 451	1.12 ± 0.12
Susquehanna drainage						
Conodoguinet	Male	ND	0	ND	ND	ND
	Female	ND	5	348 ± 160	730 ± 625	1.17 ± 0.13
Swatara up	Male	2.9 ± 0.7	13	253 ± 67	197 ± 187	1.01 ± 0.05
	Female	3.5 ± 0.6	7	364 ± 77	585 ± 317	1.11 ± 0.12
Swatara mid	Male	4.1 ± 0.6	10	382 ± 42	706 ± 248	1.23 ± 0.07
	Female	4.5 ± 0.5	10	404 ± 45	780 ± 235	1.15 ± 0.10
Swatara down	Male	3.8 ± 0.5	4	415 ± 17	878 ± 121	1.23 ± 0.08
	Female	4.5 ± 0.5	12	459 ± 27	1,098 ± 174	1.13 ± 0.10
Yellow Breeches	Male	4.3 ± 0.8	6	383 ± 6	595 ± 21	1.06 ± 0.03
	Female	4.7 ± 1.0	14	416 ± 45	768 ± 192	1.05 ± 0.10

*ND* no data

^a^All data presented as mean ± standard deviation.

^b^Condition factor (Ktl) = ((body weight − gonad weight) / length^3^) × 10^5^

Within the Susquehanna drainage, male WS collected at the upstream Swatara Creek site were shorter (*p* < 0.05) and lighter (*p* < 0.01) than those collected at the mid-Swatara site or downstream Swatara site. All others were similar in length and weight. Female WS collected at the downstream Swatara site were heavier and longer (*p* < 0.05) than those collected at the mid-site and upstream site. There were no differences among the sites in condition factor.

### White sucker—reproductive endpoints

Within the Delaware River drainage, GSI of female WS collected at the downstream Brodhead Creek site were greater than those collected at the upstream site or the Schuylkill River site. Within the Susquehanna River drainage, GSI of female WS collected at the downstream Swatara Creek site were also larger than those collected upstream, while the mid-Swatara site was not different than the other two sites. Those collected in Conodoguinet (sample size was only five) were similar to those from the upstream Swatara site, while WS collected at Yellow Breeches were similar to those collected at the downstream Swatara site (Table 7).

**Table 7:** Reproductive endpoints of white sucker collected at selected sites in the Delaware and Susquehanna River drainages in 2007–2008

Site	Female			Male		
	Sample size	GSI^a^	Vitellogenin^b^ (μg/mL)	Sample size	GSI^a^	Vitellogenin^b^ (μg/mL)
Delaware drainage						
Schuylkill	13	0.8 ± 0.3 a	9.8 ± 9.3 (9)	7	0.4 ± 0.2	14.0 ± 0.0 (1)
Brodhead upstream	8	0.8 ± 0.3 a	2,179.0 ± 4,330.0 (4)	12	0.4 ± 0.1	12.0 ± 0.0 (1)
Brodhead downstream	11	1.6 ± 0.7 b	2,237.4 ± 2,514.0 (8)	9	0.6 ± 0.3	302.6 ± 502.2 (6)
Susquehanna drainage						
Conodoguinet	5	1.1 ± 0.3 a	ND	0	ND	ND
Yellow Breeches	14	2.1 ± 0.9 b	0	6	0.6 ± 0.3	0
Swatara upstream	7	1.1 ± 0.4 a	642.7 ± 756.8 (6)	8	0.4 ± 0.1	17.3 ± 9.0 (3)
Swatara mid	10	1.4 ± 0.5 ab	631.6 ± 711.4 (8)	9	0.7 ± 0.9	49.3 ± 53.4 (9)
Swatara downstream	11	2.0 ± 0.5 b	895.0 ± 0.0 (1)	4	0.7 ± 0.1	0

*ND* no data, *GSI* gonadosomatic index

^a^Data is presented as mean ± standard deviation. Values followed by the same lowercase letters within each drainage are not significantly different (*p* > 0.05)

^b^Mean ± standard deviation of only those individuals with measurable vitellogenin. Number in parentheses indicates sample size of positive individuals

**Fig. 5:**
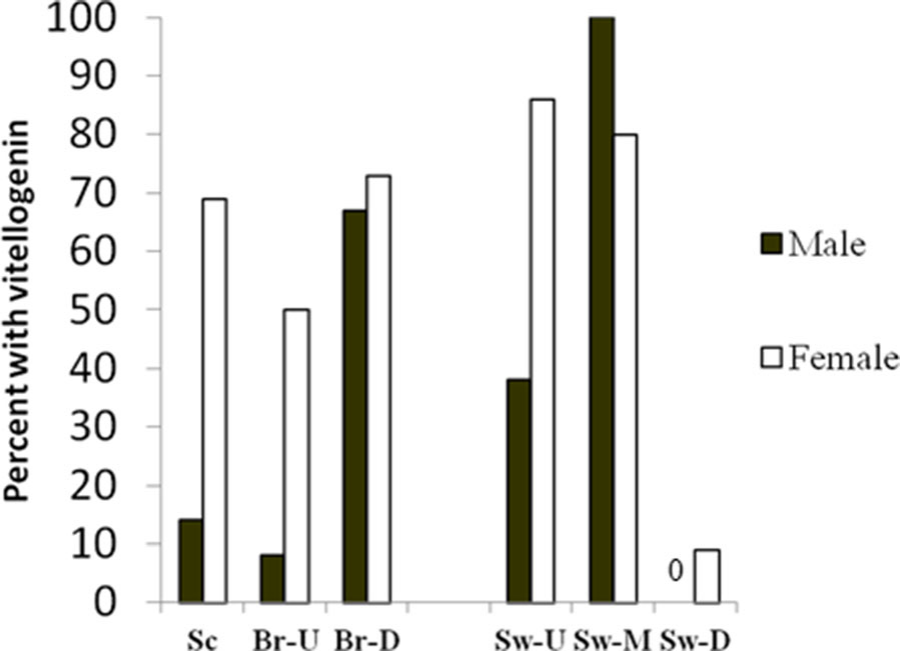
Percentage of male (*black bars*) and female (*white bars*) white sucker with measurable plasma vitellogenin collected in the Susquehanna and Delaware River drainages. Three sites, Schuykill River (*Sc*), upstream (*Br-U*) and downstream (*Br-D*) Brodhead Creek in the Delaware drainage; three sites, upstream (*Sw-U*), middle (*Sw-M*), and downstream (*SW-D*) Swatara Creek in the Susquehanna drainage

TO were not observed in WS from any site; however, no males were collected at Conodoguinet and only four at the Swatara downstream site. Vtg was detected in the plasma of male WS from the Schuylkill River, Brodhead Creek, and upstream and mid-Swatara Creek sites. None of the six males collected at Yellow Breeches or the four collected at the downstream Swatara Creek had detectable Vtg. At Brodhead Creek, there was a higher prevalence of male WS with detectable Vtg at the downstream site when compared to the upstream site. The mid-Swatara site also had a higher prevalence of males with detectable Vtg than the upstream site (Fig. 5). Both of these downstream sites showed a trend toward higher concentrations of Vtg in the male WS that had detectable concentrations; however, sample sizes were small. There was no significant difference in male GSI (Table 7), and most testes showed little to no development.

### Redhorse sucker

RHS were collected at all five sites in the Ohio River drainage and ranged in length from 180 to 605 mm. Histologically, most sucker testes showed no signs of development with the predominant cells being spermatogonia and somatic supporting cells. All but two of the RHS were 264 mm or greater which by previous studies were 3 years of age or greater and should have been mature adults (Meyer 1962). No TO were observed in RHS.

### Site comparisons

At all sites, general water quality parameters (temperature, dissolved oxygen, and pH) were within ranges not expected to adversely affect the species of interest at the time of collection. Water temperature ranged from 20.4 to 27.3 °C at sites in the Delaware drainage, from 21.4 to 28.1 °C at sites in the Susquehanna drainage, and from 25.0 to 27.2 °C at sites in the Ohio drainage. Dissolved oxygen concentrations ranged from 6.1 to 10.6 mg/L, and pH values ranged from 7.1 to 8.5 in all drainages. Watersheds above which fish were collected ranged from small drainage areas such as Yellow Breeches (534 km^2^) and Brodhead Creek (671 km^2^) to large drainage areas such as the Ohio (50,505 km^2^) and mainstem Susquehanna (29,262 km^2^) Rivers (Table 2). In general, the percentage of agricultural land use was highest throughout the Susquehanna drainage (ranging from 22 to 51 %) and the Schuylkill River (38 %) and lowest throughout the Ohio drainage (13–18 %) and Brodhead Creek (4 %). There was a significant correlation between the percent of agricultural land use in the watershed above a site and TO prevalence (rho = 0.6843, *p* = 0.0170) and severity (rho = 0.7044, *p* = 0.0129) but not with the percentage of males with Vtg (rho = 0.1926, *p* = 0.5450) when comparing the 12 sites at which SMB were collected. These watersheds also varied greatly in the number of WWTP and sewage discharges (Table 2). There was no significant relationship between the number of WWTP and TO prevalence (rho = −0.5298, *p* = 0.0794) or severity (rho = −0.8441, *p* = 0.0936) or the percentage of SMB males with Vtg (rho = 0.1617, *p* = 0.6192). No significant correlations with land use characteristics and Vtg in male WS were noted.

### Chemical analyses

Concentrations of selected hormones, as well as other chemicals known to be associated with reproductive endocrine disruption, were measured in discrete water and bed sediment samples collected at the time of the fish collections (Reif et al. 2012). Hormones detected in at least one discrete water sample are presented in Table 8. The most commonly detected hormone was estrone, found at all the Delaware and Susquehanna River sites, except the upstream Swatara site. The highest water concentration of estrone was measured at the Schuylkill site. Estrone concentrations were higher at downstream when compared to the upstream sites at Brodhead, mainstem Susquehanna, and Swatara. Androgens were only detected at Yellow Breeches, the upstream Brodhead, and downstream Susquehanna sites. No hormones were detected at any sites in the Ohio drainage (Table 8). Estrone concentrations correlated with TO prevalence (rho = 0.6530, *p* = 0.0238), severity (rho = 0.7609, *p* = 0.0055), and the percentage of male SMB with measurable plasma Vtg (rho = 0.7914, *p* = 0.0033). No correlations were noted between chemical contaminants and Vtg concentrations in male WS.

**Table 8:** Hormones (ng/L) measured in discrete water samples collected at fish health sites

Site	17-β Estradiol	Estriol	Estrone	*cis*-Androsterone	4-Androstene-3,17-dione
MDL^a^	0.05	0.05	0.05	0.05	0.05
Schuylkill	BD	BD	**2.72**	BD	BD
Brodhead up	BD	BD	*0.32*	BD	*0.40*
Brodhead down	BD	BD	**1.71**	BD	BD
Conodoguinet	BD	BD	**1.20**	BD	BD
Susquehanna up	BD	BD	*0.77*	BD	BD
Susquehanna down	BD	BD	**1.06**	BD	*0.65*
Swatara up	BD	BD	BD	BD	BD
Swatara mid	BD	BD	*0.77*	BD	BD
Swatara down	BD	BD	*0.68*	BD	BD
Juniata	*0.39*	BD	**1.31**	BD	BD
Yellow Breeches	BD	*0.23*	*0.73*	**1.53**	*0.39*
Ohio	BD	BD	BD	BD	BD
Monongahela up	BD	BD	BD	BD	BD
Monongahela down	BD	BD	BD	BD	BD
Allegheny up	BD	BD	BD	BD	BD
Allegheny down	BD	BD	BD	BD	BD

^a^The reporting level (RL) is set at least two times higher than the method detection limit (MDL). Values at or above the RL are presented in bold; those in italics are below the RL but above the MDL and are estimated (Foreman et al. 2012). Below detection (BD) denotes below the MDL

Estrone was the most commonly detected hormone in bed sediment with detections in the Schuylkill, Susquehanna upstream and downstream, Swatara mid and downstream, Juniata, and Yellow Breeches, as well as all sites in the Ohio drainage. 17β-Estradiol was also detected at the Schuylkill, Conodoguinet, Swatara mid and downstream, Juniata, Ohio, and both Monongahela sites and 17-α-estradiol at the Monongahela downstream and the Swatara downstream sites. The only sediment sample with detectable androgens (*cis*-androsterone) was the Ohio River site. Estimated concentrations of the plant sterol 3β-sitosterol were found at most sites with the highest present at the Conodoguinet, two Susquehanna, Swatara up and mid, and the two Monongahela sites (Table 9).

**Table 9:** Hormones and plant sterols (μg/kg) measured in sediment samples collected at fish health sites

Site	17-β Estradiol	17-α Estradiol	Estrone	Progesterone	*cis*-Androstene	β-Sitosterol	β-Stigmastanol
MDL^a^	0.05	0.05	0.05	0.25	0.05	363	367
Schuylkill	*0.102*	BD	**1.336**	BD	BD	*632*	*218*
Brodhead up	BD	BD	BD	BD	BD	*445*	BD
Brodhead down	BD	BD	BD	BD	BD	*441*	*70*
Conodoguinet	*0.452*	BD	BD	BD	BD	*3,072*	*833*
Susquehanna up	BD	BD	**0.444**	BD	BD	*1,058*	*265*
Susquehanna down	BD	BD	**1.336**	BD	BD	*2,184*	*53*
Swatara up	BD	BD	BD	BD	BD	*1,917*	*384*
Swatara mid	*0.569*	BD	**2.857**	*3.504*	BD	*6,625*	*1,424*
Swatara down	*0.246*	*0.061*	**1.319**	*1.015*	BD	*713*	*367*
Juniata	*0.414*	BD	**1.437**	BD	BD	*593*	BD
Yellow Breeches	BD	BD	**0.386**	BD	BD	*876*	*280*
Ohio	**0.490**	BD	**2.861**	BD	**0.78**	*889*	BD
Monongahela up	**0.222**	BD	**1.851**	BD	BD	*4,615*	*739*
Monongahela down	**0.181**	**0.082**	**0.500**	BD	BD	*2,667*	*299*
Allegheny up	BD	BD	**0.478**	BD	BD	*196*	BD
Allegheny down	BD	BD	**0.289**	BD	BD	*177*	BD

^a^The reporting level (RL) is set at least two times higher than the method detection limit (MDL). Values at or above the RL are presented in bold; those in italics are below the RL but above the MDL and are estimated, except for β-sitosterol and β-stigmastanol which are all estimated due to variable method performance (Foreman et al. 2012). Below detection (BD) denotes below the MDL

Passive water samples collected in Spring 2010 from the Juniata and Allegheny sites were analyzed for a suite of agricultural pesticides, organochlorine pesticides, PCBs, PBDEs, and hormones (Supplementary Tables 1 and 2). Both estrogens and androgens were detected at higher concentrations in the Juniata than Allegheny, as was the total estrogen equivalent quotient (total EEQ) as measured using the bioluminescent yeast estrogen (BLYES) assay (Table 10). The only pesticides detected in POCIS extracts were triazine and acetanilide herbicides, and four of the six were detected at higher concentrations in Allegheny than Juniata. Desethylatrazine was higher in Juniata, while prometon was detected in the Juniata but not in the Allegheny (Table 10). The number of detections, as well as the concentration, of all but two compounds (hexachlorobenzene, PBDE-153), was higher in the Juniata SPMD extracts, when compared to those from the Allegheny site (Supplementary Table 2).

**Table 10:** Hormones and pesticides detected in extracts of passive samplers deployed in the Juniata and Allegheny Rivers spring 2010

Chemical compounds^a^	Juniata River	Allegheny River
*Estrogenic Compounds* (ng/POCIS)		
17-α-Estradiol	**0.42**	BD
17-β-Estradiol	**1.12**	*0.18*
Estrone	**3.70**	**1.73**
Total EEQ^b^	2.94	0.78
*Androgenic Compounds* (ng/POCIS)		
*cis*-Androsterone	**0.70**	BD
4-Androstene-3,17-dione	**2.77**	**1.65**
Testosterone	**0.48**	BD
*Pesticides* estimated (ng/L) from POCIS extracts		
Desethylatrazine	*2.60*	*2.00*
Atrazine	**5.70**	**15.00**
Acetochlor	BD	**0.88**
Metolachlor	**1.70**	**4.90**
Prometon	*0.36*	BD
Simazine	BD	**3.80**

*POCIS* polar organic chemical integrative samplers, *EEQ* estrogen equivalent quotient, *BD* below detection

^a^Values at or above the RL are presented in bold; those in italics are below the RL but above the method detection limit (MDL)

^b^EEQ is the estrogen equivalent quotient as measured using the bioluminescent yeast estrogen screen (BLYES)

## Discussion

Biological indicators measured in this study demonstrated exposure to reproductive EDC in all three river drainages monitored. In particular, the presence of male SMB with TO and circulating Vtg in all three river drainages and WS males with plasma Vtg in the Susquehanna and Delaware river drainages indicates exposure to estrogenic chemicals. There were differences in the extent and severity of these effects among the drainages and sites. While TO were noted in SMB from all drainages, the overall prevalence and severity were highest in bass from the Susquehanna drainage and lowest in bass from the Ohio drainage. This is consistent with the lack of detection of estrogenic compounds in discrete water samples from the Ohio drainage in summer 2008 and the higher estrogen concentrations and higher total estrogen equivalents of the POCIS extracts from the Juniata site (Susquehanna drainage) versus the Allegheny site (Ohio drainage) in spring 2010. Interestingly, the percentage of males with Vtg and the plasma concentrations from these two sites were similar in the bass collected in the spring 2010.

SMB were the only species in which TO were observed in our study. Bass, in general, appear to be sensitive to estrogenic chemical exposure, particularly with regard to induction of TO. This was also evident in a national monitoring program conducted in nine US river basins from 1995 to 2004. TO were not observed in any of the 774 male carp collected, while 18 % of the 390 largemouth bass and 33 % of the 70 SMB collected at the same sites were observed with TO (reviewed in Hinck et al. 2009). While TO were not observed, plasma Vtg was detected in male carp at many of these sites (Hinck et al. 2008). Conversely, both WS and SMB males demonstrated Vtg production in the present study. Intersex, specifically TO, and Vtg have been widely used as biomarkers for assessing exposure to estrogenic and antiandrogenic chemicals in the aquatic environment (Heppell et al. 1995; Sumpter and Jobling 1995; Jobling et al. 1998; Blazer et al. 2007, 2012; Hinck et al. 2009; Iwanowicz et al. 2009; Jobling et al. 2009; Tanna et al. 2013). A number of studies have demonstrated that there is a poor relationship between Vtg levels and presence or severity of TO (Jobling et al. 2006; Zhao and Hu 2012), as was the case in our study. This is most likely because plasma Vtg is an indicator of recent (days to months) exposure to environmental estrogens, while induction of TO is more commonly a consequence of exposure during early life stages (Koger et al. 2000; Liney et al. 2005).

To our knowledge, there are only two reports of intersex in WS, both in WWTP effluent-dominated streams (effluent contributing up to 77 and 90 % of total stream flow) in Colorado. Intersex prevalence was 18–22 % at these sites (Woodling et al. 2006; Vadja et al. 2008). At Boulder Creek, the WWTP effluent contained a complex mixture of chemicals with maximum estrogenic equivalents of 19 to 54 ng/L at low flow. Estrogen equivalent factors (EEF) were calculated based on literature estimates for relative estrogen activity of various compounds. At certain times of the year, 17α-ethynylestradiol (EEF 1.2–3.3), 17β-estradiol (EEF 1.0), estrone (EEF 0.2–0.8), bisphenol A (EEF—7.0 × 10^5^ to 1.6 × 10^4^), and many of the nonylphenol compounds were found (Vadja et al. 2008). In our study, estrone was the only estrogenic compound consistently detected in discrete water samples during the summer sampling periods, and levels were 2.72 ng/L or less. Total estrogen equivalents, estimated from POCIS sampler extracts and the BLYES assay, as described by Alvarez et al. (2009), were 0.177 ng/L in the Juniata River and 0.048 ng/L in the Allegheny during the spring 2010 sampling. Hence, while the estrogen equivalents observed in the Susquehanna and Delaware drainages were high enough to induce Vtg production in male WS, they were apparently not high enough or did not occur at the appropriate time, to induce TO. Exposure at different life stages, seasonal differences in water or sediment concentrations, and physiological differences among species may all be important variables in understanding species differences. No TO were observed in the RHS, and the majority of the RHS testes lacked development. It is currently unknown whether the lack of testicular development in the suckers is normal for this time of year or a response to exposure to CEC.

Other studies have also demonstrated species differences in responses to estrogenic exposure. In experimental lake exposures to 17α-ethynylestradiol, TO and Vtg inductions were observed in male fathead minnows *Pimephales promelas*, while TO were not observed in WS from the same lakes, although there was induction of Vtg (Palace et al. 2009) as in our study. Differences in TO prevalence were noted even in closely related darter species (greenside darters *Etheostoma blenniodes* and rainbow darters *Etheostoma caeruleum*), suggesting species sensitivity differences (Tetreault et al. 2011). Induction of Vtg was observed in mature male fathead minnow exposed to various concentrations of poultry litter-associated contaminants, while induction only occurred in mummichog *Fundulus heteroclitus* at the highest concentration and sheepshead minnow *Cyprinodon variegatus* were unresponsive. Interestingly, all three species produced Vtg in response to 17β-estradiol (Yonkos et al. 2010), suggesting that fathead minnows may respond to other estrogenic compounds present in poultry litter which the other species did not.

It is unclear whether there are certain chemicals that SMB are especially sensitive to or if the apparent increased sensitivity to induction of TO is a factor of life history and differential exposure at sensitive life stages when compared to WS. Higher numbers and concentrations of estrogenic compounds were noted in sediment when compared to water. It is possible that the species difference in TO presence may be a result of spawning behavior/substrate. In streams, SMB males prepare nests about two to four inches deep by sweeping the silt, sand, and fine gravel with the caudal fin, leaving a bed of coarse gravel and rock. Nests are kept clean before spawning; however, after spawning, nests may become covered with a coating of fine sediment within a few days. Eggs hatch about 3 days after spawning, and the newly hatched fry drop down into the gravel where they remain for approximately six more days (Pflieger 1966). Hence, fry are in direct contact with sediment during the most sensitive stage for effects on sexual development and induction of TO (Koger et al. 2000; Krisfalusi and Nagler 2000; Liney et al. 2005). Conversely, suckers do not prepare nests but are bottom spawners in swift water over gravel. Eggs are slightly adhesive and attach to rocks or bottom gravel. Some populations make spawning runs from larger streams/rivers into gravel-covered smaller streams with medium-sized gravel in riffle zones (Corbett and Powles 1986; Curry and Spacie 1984). Black redhorse and golden redhorse have been reported to spawn after WS and show little or no movement for spawning (Curry and Spacie 1984; Meyer 1962). Hence, there may be less direct contact of sucker eggs or fry with sediment-associated EDCs.

Estrone was the most commonly detected hormone in both water and sediment samples. It is a natural hormone, excreted by numerous vertebrate groups. It is also the primary metabolite of α- and β-estradiol oxidation (Hanselman et al. 2003), and hence estrone concentrations are often higher than estradiol in the environment (Kolpin et al. 2002; Vajda et al. 2008). Estrone is commonly found in WWTP effluent (Björkblom et al. 2008; Vajda et al. 2008) as well as various animal manures including poultry (Andaluri et al. 2012), bovine (Andaluri et al. 2012; Kolodziej et al. 2004), swine (Shore and Shemesh 2003), and aquaculture/hatchery effluent (Kolodziej et al. 2004). Estrone is a potent EDC, with reported relative estrogenic equivalents (with β-estradiol equal to 1.0) of 0.2 to 0.8, depending on the assay (Vajda et al. 2008; Van den Belt et al. 2004) and perhaps the fish species. Estrone had equal potency to 17β-estradiol for Vtg induction in adult female zebrafish *Danio rerio* (Van den Belt et al. 2004). However, estrone induced plasma Vtg at lower exposure concentrations (31.8 ng/L) than 17β-estradiol (100 ng/L) in adult male fathead minnows (Panter et al. 1998). It is interesting to note that TO prevalence and severity and percent of male SMB with Vtg all showed a significant correlation with estrone concentrations in both discrete water and sediment samples collected at the time of fish collections. It is currently unknown how SMB or suckers may differ in their response to exposure to individual estrogens.

The sources of estrogenic chemicals are most likely both human waste (WWTP effluent, sewage discharges) and agricultural runoff. While the prevalence of male SMB with TO was not higher at sites immediately downstream of WWTPs, the severity of TO and percentage of males with plasma Vtg generally increased at downstream sites. This is similar to observations on SMB collected at sites upstream and downstream of WWTPs in two tributaries of the Potomac River (Iwanowicz et al. 2009). However, “upstream” sites in this study, as well as the Potomac study, were not headwater sites, and consequently, other WWTP effluents entered the rivers/streams further upstream. Increased TO prevalence downstream of a second WWTP on a Canadian River was suggested to be due to cumulative impacts from both effluents. However, in 6.3 km downstream of the second WWTP, the occurrence of TO dropped to reference levels (Tetreault et al. 2011; Tanna et al. 2013). Many factors such as flow, the proportion the effluent contributes to total flow, season, type of treatment the facilities utilize, and population served can all influence the impact of WWTP effluents on the aquatic environment. Further research with more sites along a particular stream, and larger sample sizes, collected during multiple seasons/flow regimes would be necessary to fully understand the role of WWTP effluent on the observed biological effects within these drainages.

In this study, as well as previous studies in the Potomac drainage, TO prevalence and severity correlated with the percent of agricultural land use in the catchment above the collection site. An assessment of six sites in the Potomac drainage and one out-of-basin reference site (Ohio drainage) indicated a positive correlation of TO prevalence with agricultural use and number of animals (in animal feeding operations) in the catchments above the sites, while severity positively correlated with those land-use factors as well as with WWTP flow, total animal feeding operations, and poultry houses (Blazer et al. 2012). In terms of actual chemical concentrations, only atrazine in discrete water samples collected during the spring spawning period correlated with TO. Interestingly, no hormones were detected in the discrete water samples collected during the spring sampling at the Potomac sites. Conversely, in bed sediment collected in SMB nesting areas, β-sitosterol, β-stigmastanol, and trans-nonachlor correlated with TO. A significant positive relationship (rho = 0.986, *p* < 0.001) was observed between TO and total hormone/sterol bed sediment concentrations (Kolpin et al. 2013). The phytosterol, β-sitosterol, has been shown to bind to estrogen receptor β (Gutendorf and Westendorf 2001) and to be estrogenic (Tremblay and Van 1999). The role of phytoestrogens and plant sterols in induction of TO and Vtg in male fishes needs further research.

The studies to date in the Potomac and Susquehanna drainages provide additional weight of evidence for the influence of nonpoint sources, particularly agricultural sources, on the health of resident fish populations. Estrogens are known to enter the aquatic environment through runoff or leachate from application of manure from animal feeding operations (Kjaer et al. 2007; Matthiessen et al. 2006), directly from grazing animals adjacent or in stream/rivers (Kolodziej and Sedlak 2007), and application of human biosolids to agricultural field (Andaluri et al. 2012). A study in small watersheds of the Shenandoah River (Potomac drainage) indicated no significant relationship between total estrogen equivalents (during periods of high or low stream flow) with WWTP discharges but a strong relationship with density of animal feeding operations (Ciparis et al. 2012). Characterization of sources and timing of exposure at particular sites are necessary for better management of the land-use practices contributing to adverse fish health effects. There are studies that suggest exposure to the EDC that induce TO and plasma Vtg in male fishes that can have population-level consequences due to reproductive impairments (Harris et al. 2011; Kidd et al. 2007; Miller et al. 2007). There is also increasing evidence that these same chemicals may affect the immune system and disease resistance (Iwanowicz and Ottinger 2009; Liney et al. 2006; Milla et al. 2011) and hence could have population-level effects through increased mortality, both acute and chronic, in addition to reproductive effects. There is currently no direct evidence linking the disease and mortality observed in SMB and other fishes from the Potomac and Susquehanna Rivers to estrogenic chemicals. However, the co-occurrence of a high prevalence of skin lesions and fish mortality with the high prevalence of TO and Vtg in male fishes suggests a connection and requires further study.

In conclusion, evidence of exposure to estrogenic contaminants was observed in all three major river drainages within Pennsylvania and in two fish species. TO were not noted in any of the suckers; however, Vtg induction occurred in male WS. A higher prevalence and severity of TO were noted in SMB from the Susquehanna drainage when compared to SMB from the Ohio drainage, which may be associated with both land use influences and flow regimes, which differ greatly between the river systems.

## Electronic supplementary material

Below is the link to the electronic supplementary material.

ESM 1 (DOCX 26 kb)

ESM 2 (DOCX 27 kb)
