# Supplementary material for: Reproductive health indicators of fishes from Pennsylvania watersheds: association with chemicals of emerging concern
Source: Environ Monit Assess. 2014 Jun 17;186(10):6471–91. doi: 10.1007/s10661-014-3868-5 (PMC4149881; doi:10.1007/s10661-014-3868-5)
Supplement: Supplementary file 1 — (DOCX 26 kb) [file 10661_2014_3868_MOESM1_ESM.docx]

Online Resource 1

Supplementary Table 1. The Method Detection Limit (MDL) and Reporting Level (RL) for Pesticides and Hormones Measured in POCIS Extracts

| Pesticides | MDL  ng/L | RL  ng/L | Hormones | MDL  ng/POCIS |
| --- | --- | --- | --- | --- |
| Desisopropylatrazine | 2.70 | 13.0 | Bisphenol A | 100.0 |
| Desethylatrazine | 1.10 | 5.70 | Diethylstilbestrol | 0.4 |
| Trifluralin | 1.20 | 5.80 | cis-Androsterone | 0.4 |
| Atraton | 0.23 | 1.20 | Epitestosterone | 2.0 |
| Simazine | 0.53 | 2.70 | 17-α-Estradiol | 0.4 |
| Prometon | 0.13 | 0.65 | Dihydrotestosterone | 2.0 |
| Atrazine | 0.47 | 1.20 | 4-Androstene-3,17-dione | 0.4 |
| Propazine | 0.26 | 1.30 | Estrone | 0.4 |
| Terbuthylazine | 0.21 | 1.10 | 17-β-Estradiol | 0.4 |
| Fonofos | 1.00 | 5.20 | Testosterone | 0.4 |
| Diazinon | 0.14 | 0.69 | Equilin | 2.0 |
| Metribuzin | 0.70 | 3.50 | 11-Ketotestosterone | 1.0 |
| Acetochlor | 0.15 | 0.73 | Norethindrone | 0.4 |
| Methyl Parathion | 2.40 | 12.0 | Mestranol | 0.4 |
| Simetryn | 0.16 | 0.78 | Equilenin | 1.0 |
| Alachlor | 0.17 | 0.87 | 17-α-ethynylestradiol | 0.4 |
| Ametryn | 0.17 | 0.87 | Estriol | 1.0 |
| Prometryn | 0.20 | 1.00 | Progesterone | 4.0 |
| Terbutryn | 0.18 | 0.91 | 3-β-Coprostanol | 200.0 |
| Malathion | 59.0 | 290.0 | Cholesterol | 200.0 |
| Metolachlor | 0.13 | 0.67 |  |  |
| Chlorpyrifos | 2.20 | 11.0 |  |  |
| Dacthal | 1.00 | 5.10 |  |  |
| Pendimethalin | 0.23 | 1.10 |  |  |
| Fipronil | 1.10 | 5.70 |  |  |
