# Supplementary material for: Reproductive health indicators of fishes from Pennsylvania watersheds: association with chemicals of emerging concern
Source: Environ Monit Assess. 2014 Jun 17;186(10):6471–91. doi: 10.1007/s10661-014-3868-5 (PMC4149881; doi:10.1007/s10661-014-3868-5)
Supplement: Supplementary file 2 — (DOCX 27 kb) [file 10661_2014_3868_MOESM2_ESM.docx]

Online Resource 2

Supplementary Table 2. Organochlorine Pesticides, Polychlorinated Biphenyls and Polybrominated Diphenyl Ethers Measured in SPMD Extracts

| Compound | Juniata River  pg/L | Allegheny River  pg/L | MDL^1^  pg/L | RL^1^  pg/L |
| --- | --- | --- | --- | --- |
| Trifluralin | BD^1^ | BD | 2.8 | 14.0 |
| Hexachlorobenzene | **16.0** | **23.0** | 1.1 | 5.6 |
| Pentachloroanisole | **95.0** | **62.0** | 10.0 | 27.0 |
| α-Hexachlorocyclohexane | **59.0** | BD | 4.9 | 25.0 |
| Lindane | BD | BD | 7.0 | 35.0 |
| β- Hexachlorocyclohexane | BD | BD | 4.9 | 25.0 |
| Heptachlor | BD | BD | 1.1 | 5.7 |
| δ- Hexachlorocyclohexane | BD | BD | 32.0 | 100 |
| Dacthal | BD | BD | 110.0 | 310 |
| Chlorpyrifos | *69.0* | BD | 67.0 | 200 |
| Oxychlordane | BD | BD | 35.0 | 100 |
| Heptachlor epoxide | BD | BD | 70.0 | 180 |
| trans-Chlordane | *46.0* | BD | 22.0 | 59.0 |
| trans-Nonachlor | **46.0** | **10.0** | 1.4 | 6.8 |
| o,p’-DDE | BD | BD | 39.0 | 120 |
| cis-Chlordane | **59.0** | BD | 17.0 | 46.0 |
| Endosulfan | **140.0** | BD | 22.0 | 110 |
| p,p’-DDE | BD | BD | 76.0 | 160 |
| Dieldrin | BD | BD | 77.0 | 170 |
| o,p’-DDD | BD | BD | 46.0 | 120 |
| Endrin | BD | BD | 1.5 | 7.7 |
| cis-Nonachlor | BD | BD | 37.0 | 85.0 |
| o.p’-DDT | BD | BD | 1.1 | 5.5 |
| p,p’-DDD | *23.0* | BD | 19.0 | 58.0 |
| Endosulfan-II | *830* | BD | 680 | 2000 |
| p,p’-DDT | BD | BD | 84.0 | 140 |
| Endosulfan sulfate | BD | BD | 32.0 | 160 |
| p,p’-Methoxychlor | BD | BD | 1.6 | 7.8 |
| Mirex | BD | BD | 1.8 | 9.1 |
| cis-Permethrin | BD | BD | 220 | 510 |
| trans-Permethrin | BD | BD | 190 | 580 |
| Total PCBs | *620* | *320* | 270 | 1400 |
| PBDE-28 | BD | BD | 40.0 | 110 |
| PBDE-47 | BD | BD | 88.0 | 210 |
| PBDE-99 | *28.0* | BD | 28.0 | 68.0 |
| PBDE-100 | *12.0* | BD | 7.9 | 18.0 |
| PBDE-153 | **11.0** | **14.0** | 3.6 | 8.9 |

^1^BD is below method detection limit (MDL), bold values are greater than the method reporting limit (MRL) and italic values are between MDL and MRL.
